# Penicillium on the Iris! An Unusual Presentation and Cause of Postoperative Anterior Uveitis

**DOI:** 10.7759/cureus.5343

**Published:** 2019-08-07

**Authors:** Vijaya Jojo, Minakshi Gupta, Bharti Sharma, Poonam Singh, Nitin Dhira

**Affiliations:** 1 Ophthalmology, Tata Main Hospital, Jamshedpur, IND; 2 Microbiology, Tata Main Hospital, Jamshedpur, IND; 3 Ophthalmology, Tata Memorial Hospital, Jamshedpur, IND; 4 Ophthalmology, Jamshedpur Eye Hospital, Jamshedpur, IND

**Keywords:** penicillium, iris lesion, postoperative uveitis

## Abstract

Intraocular fungal infections may not present solely as postoperative anterior uveitis or as a focal anterior segment lesion. The present study describes a 50-year-old woman with well-controlled diabetes who presented with postoperative anterior uveitis three months after uncomplicated cataract surgery. A fuzzy lesion was observed on her iris. The patient underwent an anterior chamber wash and removal of the lesion, followed by intracameral treatment with voriconazole. Culture of the lesion showed that it was a species of Penicillium. The patient has remained stable after treatment. Three aspects of this case were unusual: a fungal lesion of unusual etiology and location, inflammation restricted to the anterior segment despite a fungal background, and the excellent response to treatment with a very favorable outcome.

## Introduction

Postoperative fungal infections of the eye have been reported to present as endogenous endophthalmitis with an incidence ranging from 16.7% to 70% [1-2]. Endogenous endophthalmitis occurs especially in patients with drug-induced immunosuppression, organ transplantation, or a nidus for infection such as an indwelling catheter. The most common fungal pathogens identified in patients with such infections are Candida and Aspergillus [2-3]. The present study describes a 50-year-old woman with well-controlled diabetes who presented with postoperative anterior uveitis along with an unusual growth on the iris, three months after uncomplicated cataract surgery. The pathogen identified here was Penicillium Spp. and, to our knowledge has not been reported to date.

## Case presentation

**Figure 1:**
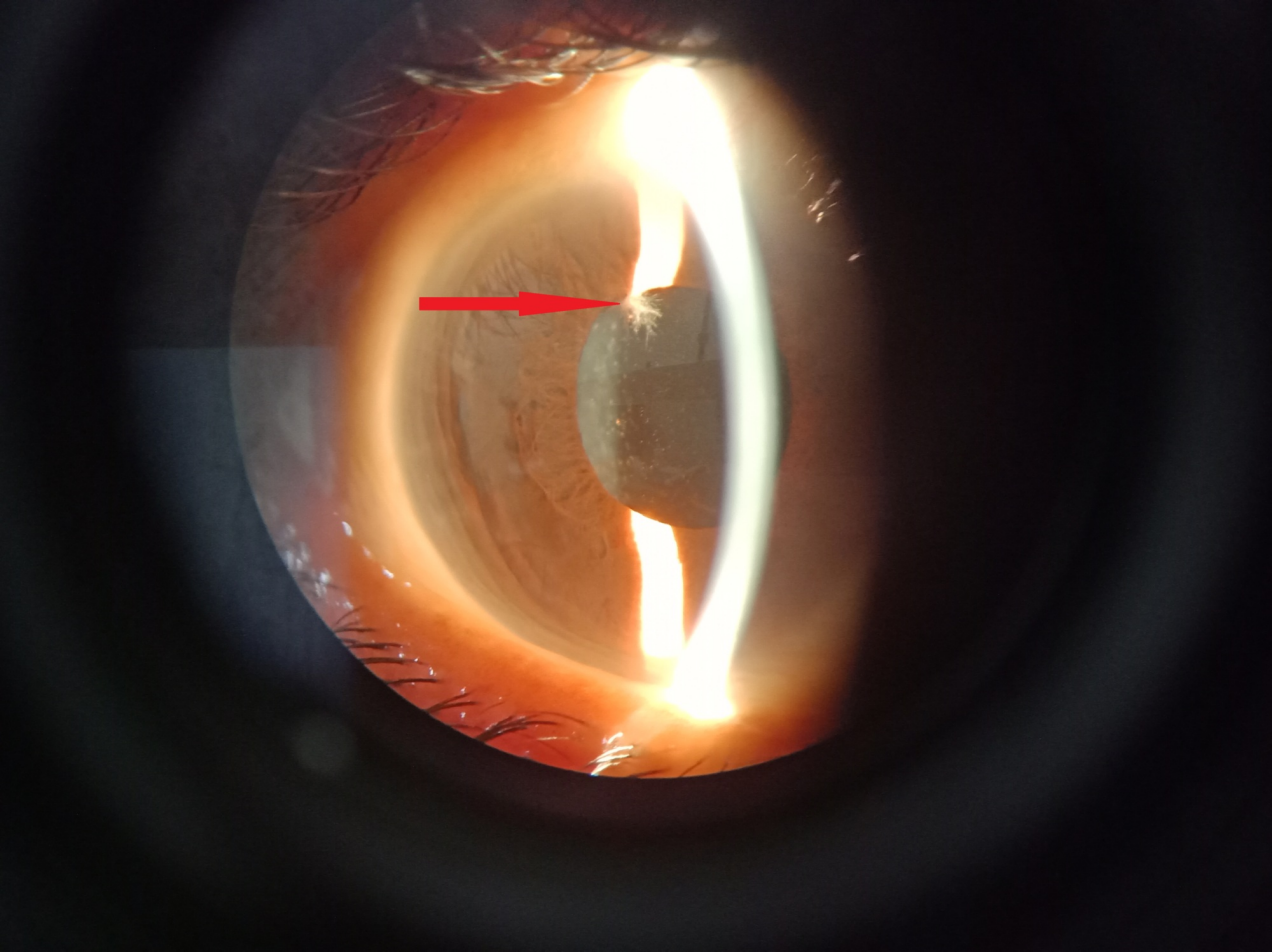
Hairy lesion on the iris.

A 50-year-old woman underwent a cataract operation on her right eye in January 2019. She had diabetes but was well-controlled on oral hypoglycemic agents. Following successful surgery, a refractive correction was prescribed, and she was discharged from the cataract clinic after one month with a best-corrected visual acuity of 20/20. In late March 2019, the patient presented to the ED with pain, photophobia, and decreased vision. On examination, her visual acuity was 20/80, her intraocular pressure was normal, and dilated fundus examination showed a good fundal glow without any evidence of posterior segment pathology. The anterior segment of her right eye showed evidence of acute anterior nongranulomatous uveitis, and a small but noticeable lesion with hairy margins was observed at the 11 o'clock position on the anterior surface of the iris close to the pupillary margin (Figure 1).

The appearance of the lesion suggested a possible fungal origin. The patient was immediately subjected to an anterior chamber wash. The lesion was gripped with tooth forceps, removed from the iris and plated onto sabouraud dextrose agar (SDA). An aqueous tap was also plated onto standard chocolate and blood agar. The patient was administered an intracameral injection of 0.1 mL containing 100 µg voriconazole using an insulin syringe.

**Figure 2:**
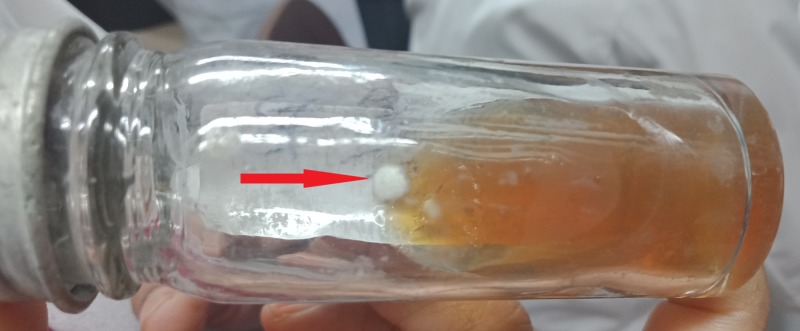
SDA culture specimen on day five. SDA, sabouraud dextrose agar.

**Figure 3:**
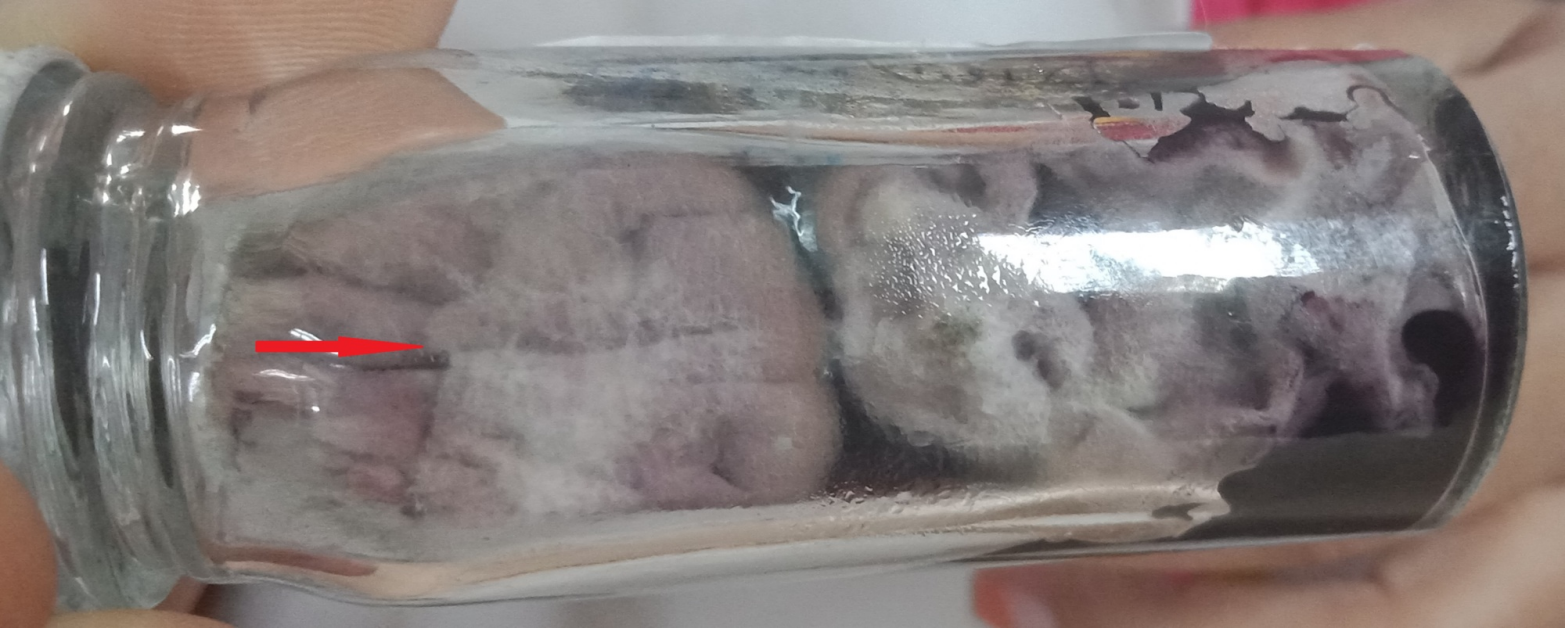
Culture specimen on day 14.

The examination the next day showed that the eye was mildly inflamed but less so than before the treatment. She was subsequently treated with topical steroids and topical 1% voriconazole drops, six times daily. Culture of the fungus on SDA on day five showed that the pathogen was Penicillium Spp. (Figure 2),

which was further confirmed on day 14 (Figure 3).

**Figure 4:**
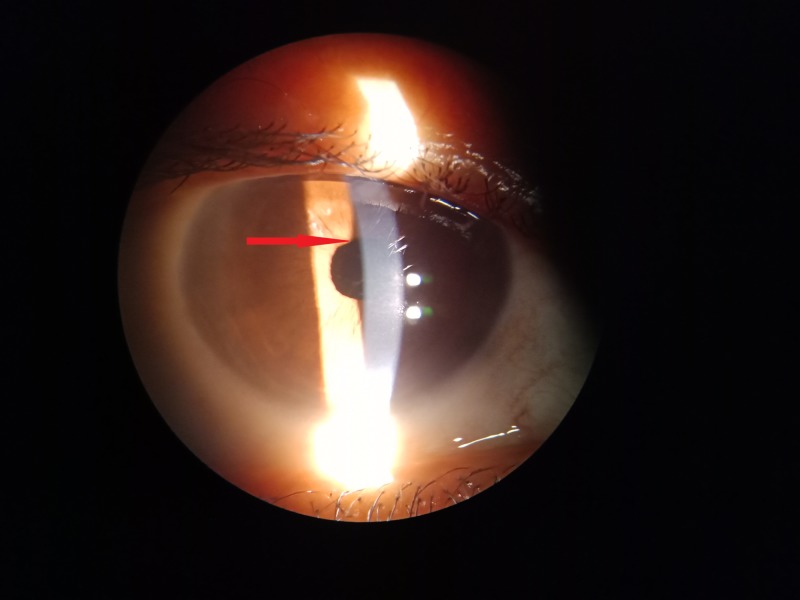
Noninflamed eye at three months postoperative with patches of iris atrophy at the initial site of lesion.

As her blood tests showed borderline increases in liver transaminases and an absence of any other risk factors for fungemia, she was not treated with any systemic antifungal agents. The patient was gradually tapered off both steroids and voriconazole over the next six to eight weeks. At three months, the patient had a best-corrected visual acuity of 20/20 and a noninflamed eye (Figure 4).

## Discussion

Postoperative fungal infections have been reported to give rise to endophthalmitis, with outcomes that depend on the infecting organism and delay in presentation [4]. Although our patient had diabetes, her disease was well-controlled, and she had no other predisposition to a fungal organism. Moreover, the infection in this patient showed an uncommon clinical presentation, being an extremely well-localized lesion with no involvement of the posterior chamber of the eye.

The pathogen identified in this patient was Penicillium Spp., a ubiquitous organism present in the environment. Although patients have been reported with keratitis and postoperative endophthalmitis due to Penicillium, none of these patients showed a similar presentation to ours [5-7].

As the pathogen was unusual, treatment was unclear. Voriconazole has shown broad fungistatic and fungicidal activities, with topical preparations achieving sufficiently high concentrations in the aqueous and vitreous humor-concentrations higher than those achieved by oral formulations [8]. The clinical suspicion of possible fungal pathology, later confirmed by culture, led us on to continue with this line of treatment. Prompt identification and management of the pathogen ensured that the patient recovered completely with a good visual outcome.

## Conclusions

Fungal infections following cataract surgery are uncommon, more so the presentation and the organism in our case were unusual. The very rare clinical signs, time of presentation, and a strong suspicion prompted us to treat this as a post-operative fungal infection. The removal of the lesion along with a combination of intracameral and topical antifungal medications ensured that the patient achieved a complete cure. The well-localized lesion and confirmation of Penicillium Spp. by culture reports encouraged us to report this clinical scenario, which we believe is the first of its kind. Findings in this patient suggest that a thorough examination and a high index of suspicion are needed for proper diagnosis and treatment. Although rare pathogens are challenging to manage, appropriate identification and treatment can ensure successful outcomes.
